# Support vector regression algorithm modeling to predict the parturition date of small - to medium-sized dogs using maternal weight and fetal biparietal diameter

**DOI:** 10.14202/vetworld.2021.829-834

**Published:** 2021-04-02

**Authors:** Thanida Sananmuang, Kanchanarat Mankong, Suppawiwat Ponglowhapan, Kaj Chokeshaiusaha

**Affiliations:** 1 Faculty of Veterinary Medicine, Rajamangala University of Technology Tawan-OK, Chonburi, Thailand; 2 Smile Dog Small Animal Hospital, Chonburi, Thailand; 3 Department of Obstetrics, Gynaecology and Reproduction, Research Unit of Obstetrics and Reproduction in Animals, Faculty of Veterinary Science, Chulalongkorn University, Bangkok, Thailand

**Keywords:** biparietal diameter, dog size, prediction accuracy, support vector regression

## Abstract

**Background and Aim:**

Fetal biparietal diameter (BPD) is a feasible parameter to predict canine parturition date due to its inverted correlation with days before parturition (DBP). Although such a relationship is generally described using a simple linear regression (SLR) model, the imprecision of this model in predicting the parturition date in small- to medium-sized dogs is a common problem among veterinarian practitioners. Support vector regression (SVR) is a useful machine learning model for prediction. This study aimed to compare the accuracy of SVR with that of SLR in predicting DBP.

**Materials and Methods:**

After measuring 101 BPDs in 35 small- to medium-sized pregnant bitches, we fitted the data to the routine SLR model and the SVR model using three different kernel functions, radial basis function SVR, linear SVR, and polynomial SVR. The predicted DBP acquired from each model was further utilized for calculating the coefficient of determination (R2), mean absolute error, and mean squared error scores for determining the prediction accuracy.

**Results:**

All SVR models were more accurate than the SLR model at predicting DBP. The linear and polynomial SVRs were identified as the two most accurate models (p<0.01).

**Conclusion:**

With available machine learning software, linear and polynomial SVRs can be applied to predicting DBP in small- to medium-sized pregnant bitches.

## Introduction

Parturition is a critical process in clinical animal obstetrics. In pregnant bitches, timely assisted delivery assures healthy puppies for the owners and may prevent the tragic loss of pets. Thus, accurately predicting parturition dates are crucial in canine obstetric care [1-3]. Several techniques based on a variety of evaluation methods have been invented for predicting parturition [3]. Transabdominal ultrasonography is a practical technique performed by veterinarians for parturition date prediction [1,2]. This technique can be utilized to measure four major parameters: Embryonic vesicle diameter, crown-rump length, body diameter, and biparietal diameter (BPD) [1-4].

BPD is regarded as an accurate parameter for predicting parturition dates in the second half of gestation [3,5]. BPD facilitates veterinarian planning for the impending parturition and is a crucial measurement for pregnancy follow-up [2,5]. A simple linear regression (SLR) model has been used to describe the relationship between BPD and days before parturition (DBP) [1,2]. Despite the routine application of the SLR model in real practice, requirements for size-specific formulas indicate considerable limitations to this model in predicting DBP based on BPD [2,3,6,7].

Inaccurate DBP predictions using the SLR model usually occur in small- to medium-sized pregnant bitches (maternal weights 1-25 kg) due to several factors, including gestation time and large variations in BPD among dog breeds [2,3,7,8]. Of note, several popular dog breeds of these sizes are at high risk of dystocia, the clinical condition of difficult labor [9,10]. Accurate DBP predictions are crucial in these dog breeds to manage laboring support or elective cesarean deliveries. Ambiguous correction days for the DBP calculations (±1-2 days) [2,3,7] and breed-specific models [11] were suggested to improve the accuracy of the SLR model. However, these limitations highlight the need for an improved regression model to optimize the prediction of DBP.

Advances in machine learning algorithms brought about innovations in predictive models for medical science. Support vector regression (SVR), a regression model that uses similar principles to support vector machine (SVM) classification, has been successfully applied to a variety of fields [12-14]. Unlike SLR, SVR can incorporate non-linear relationships of several variables by applying different kernel functions; the mathematical functions used to transform data from non-linear space to linear space [15]. A suitable kernel function can reveal the underlying relationship of the input data to the expected output. Similar to SVM, the three popular inner product-based kernels are linear, polynomial, and radial basis function (Rbf) kernels. These kernel functions are productive in most conditions, making them reasonable targets for the development of a new predictive model [16].

Because SVR is flexible at dealing with undefined distributional properties of underlying variables, this model is an appealing candidate model to capture unknown geometric correlations among weight, BPD, and DBP to improve DBP prediction accuracy in small- and medium-sized pregnant bitches. The current study aimed to evaluate SVR as an alternative model for predicting DBP using maternal body weight and BPD data.

## Materials and Methods

### Ethical approval

This study used only anonymous data already collected and presented in the database of an animal hospital. There was no experiment with animals included in this study.

### Experimental design

**Figure-1:**
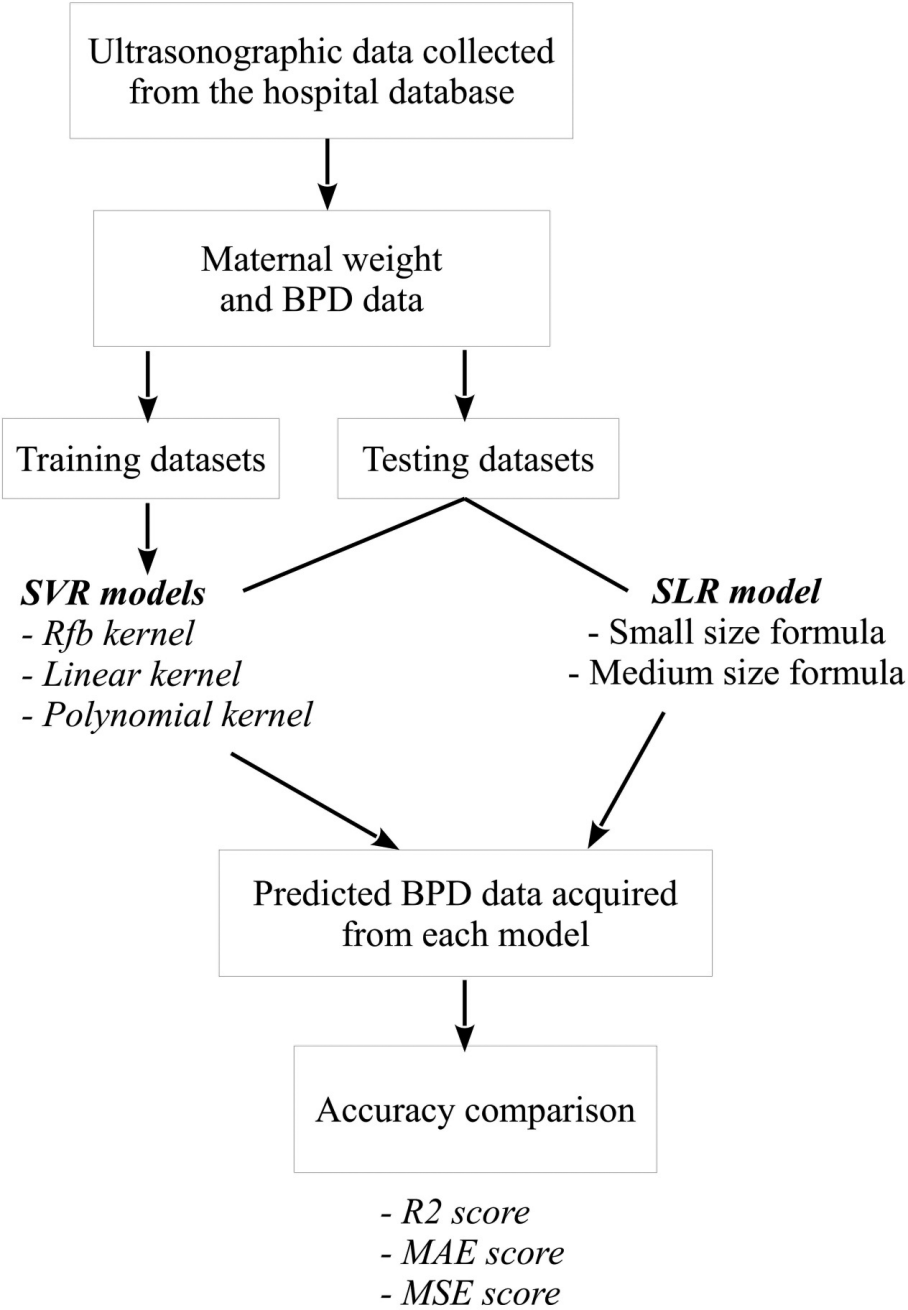
Experimental flowchart. Maternal weight and fetal biparietal diameter (BPD) data were collected from a local animal hospital. Training datasets and testing datasets were generated for training support vector regression (SVR) models and testing their performances comparing to those acquired from conventional simple linear regression (SLR) models. The total of 3 SVR models was obtained as follows: Radial basis function SVR (Rbf SVR), linear SVR, and polynomial SVR. Predicted DBPs of testing datasets were calculated from trained SVR models and conventional SLR models. Accuracy among these models was compared using bootstrap coefficient of determination (R2), mean absolute error (MAE), and mean squared error (MSE) scores acquired among regression models.

All data used in this study were anonymously collected from the database (1/1/2018 - 28/12/2018) of small- to medium-sized bitches presented for routine pregnancy follow-up at a local animal hospital in Chonburi Province, Thailand. The analytical process of this study is summarized in Figure-1. Each time a pregnant bitch presented at the hospital, the dog’s weight (maternal weight) and mean fetal BPDs of all fetuses (five repeats for each fetus) were determined. All pregnant bitches presented for pregnancy follow-up from 1 to 26 DBP.

Python programming language was used for all statistical analyses. The data were divided into training (98 samples) and testing (40 samples) datasets using our in-house Python function to ensure all dog breeds were presented in both datasets. With acquired testing datasets, the expected DBP was calculated using the SLR model compatible with the dog size. In contrast, the k-fold cross-validation method was applied to the training datasets (5-fold cross-validation repeated 100 times independently) for training SVR models using the three different kernel functions, Rbf SVR, linear function (linear SVR), and polynomial function (polynomial SVR). Parameter optimization of each model was achieved using the Optunity software package (https://optunity.readthedocs.io/en/latest/#). The performance of each optimized SVR model was subsequently verified in testing datasets. To identify the most accurate model, comparisons of bootstrap coefficient of determination (R2), mean absolute error (MAE), and mean squared error (MSE) scores acquired among regression models were performed using one-way analysis of variance (ANOVA) with *post hoc* Tukey honestly significant difference (HSD).

### Programming environment and python packages

The open-source web application, Jupyter Notebook, was utilized to create and run Python coding. All Python packages required for the study are listed in Table-1.

**Table-1:** Python and R packages.

Programming	Packages	Usages
Python3	matplotlib	Distribution plot and graph plot
	numpy	Data array management; R2, MAE, and MSE calculation
	optunity	SVR parameter optimization
	pandas	Dataframe management
	sklearn	Model fitting and prediction

### Animals

A total of 35 pregnant bitches (2-5 years old) presented as patients for pregnancy diagnosis. BPD examinations acquired from the bitches (n=138) were categorized according to dog sizes into small size (1-10 kg) (n=75) and medium size (>10-25 kg) (n=63). All dog breeds presented for diagnosis were alphabetically ordered as follows: Bulldog (n=5), Bully (n=8), Chihuahua (n=34), Corgi (n=15), French bulldog (n=34), mixed breed (n=14), and Pomeranian (n=28). All dog breeds were included in both training (n=98) and testing (n=38) datasets after division. All bitches included in this study either delivered naturally or through elective cesarean delivery. The major criteria for cesarean were observable before delivery, including panting, nesting, laboring, and showing fetal heart rate <180 bmp [17,18]. The cesarean delivery operations were both planned and emergent, because the false predictions obtained from the conventional SLR model were observable. Of note, pregnancies with one puppy were also included in the current study. All delivered puppies were alive after a 1-week follow-up.

### BPD examination

Ultrasonography for pregnancy diagnosis was performed to determine fetal BPD. All bitches were examined in dorsal recumbency without sedation. Transmission gel was applied directly to the dogs after hair clipping. Real-time ultrasound images were produced using a 7.5 MHz transducer (LOGIQ V3, General Electronic Company, USA). After locating the uterus, at least two fetuses from two opposite uterine horns were evaluated. The techniques for measuring BPD are described by Lenard *et al*. [19]. Briefly, BPD obtained from two adjacent fetuses was used to distinguish the different fetuses; the average BPD was represented. Selected fetuses were normally adjacent to one another to avoid confusion from the same fetus measurements. In this study, BPD was measured from the outer edge of the proximal calvarial wall to the outer edge of the distal calvarial wall, at the widest part of the skull [20].

### Statistical analysis

#### K-fold cross-validation

To avoid the overfitting problem of modeling, training datasets and testing datasets were generated for SVR parameter optimization as described in the Optunity package [21]. Partitioning of data samples into such datasets was accomplished by applying the stratified 5-fold cross-validation(1000 iterations). In brief, the original training datasets were randomly partitioned into five equal-sized subsamples. Of the five subsamples, a single subsample was retained for each iterated SVR model. The remaining subsamples of each iteration were used as training data for further SVR optimization.

#### SLR

The reported SLR model formulas were used to evaluate the relationships between BPD and DBP [3]. The adjusted intercept coefficient value (Coef) and the first-order coefficient value (inter) for each formula according to the dog size are summarized in Table-2. SLR model fitting and DBP predictions were accomplished using the “scikit-learn” package [22].

**Table 2:** Models and corresponding parameters used in this study.

Model	Abbreviation	Optimized parameters	
Simple linear regression	SLR	Small-sized dog	^a^Coef=(−1/0.61), ^b^inter=(25.11/0.61)
		Medium-sized dog	^a^Coef=(−1/0.7), ^b^inter=(29.18/0.7)
Support vector regression using radial basis function	Rbf SVR	^c^C=(57.35652343749997), ^d^gamma=(0.044921875000007105)	
Support vector regression using linear function	Linear SVR	^c^C=(2.695418693334262)	
Support vector regression using polynomial function	Polynomial SVR	^c^C=(15862.3046875), ^e^coef0=(0.1103515625), ^f^degree=(2.1025390625)

a coef=Coefficients for the linear regression,

b inter=Intercept for linear regression,

c C=Regularization parameter,

d gamma=Kernel coefficient,

e coef0=Independent term,

f degree=Degree of the polynomial kernel function

#### Support vector regression

Because this study mainly aimed to demonstrate procedures for practitioners, the focus was on describing major differences between SVR and SLR using their concepts. SLR aimed to rigidly minimize the error rate produced by the predicted values on the line, whereas SVR flexibly provided an acceptable margin for an appropriate line or even plane fit as many data points as possible. SVR thus aimed to fit the error rate to include as many data points as possible within a certain threshold. This was achieved by setting a decision boundary at e (epsilon) distance from the optimal hyperplane, the decision surfaces produced by predicted values. To extend such a concept for non-linear decision surfaces, the kernel functions were applied to transform the original data to map into a new space. In this study, the required parameters for each kernel function were initially optimized in the generated training datasets using the Optunity package (Table-2). The Optunity package was utilized in this study because of its available functions for tuning all hyperparameters in SVR to optimize each kernel function [21]. SVR model fitting and DBP predictions were accomplished by utilization of the “scikit-learn” package, which provided a variety of functions for the machine learning study [22]. In this study, the SVR models produced using radial basis, linear, and polynomial kernel functions are addressed as Rbf SVR, linear SVR, and polynomial SVR, respectively.

#### Coefficient of determination (R2), MAE, and MSE scores

The performance, that is, how close the predicted DBP compared with the real DBP of each model, was determined by calculating three scores: The coefficient of determination (R2), MAE, and MSE scores. These scores were obtained from “sklearn.metrics.r2_score,” “mean_absolute_error,” and “sklearn.metrics.mean_squared_error” functions provided in the “scikit-learn” package. In brief, a high R2 (near 1.0), low MAE, and low MSE scores imply good prediction accuracy of a model. R2, MAE, and MSE scores, which were calculated from 1000 resamples of DBP results for each regression model, were acquired using the bootstrapping method. The scores acquired among models were then compared with one another using one-way ANOVA with *post hoc* Tukey HSD tests (p<0.01).

## Results

### BPD and DBP acquired from small- and medium-sized pregnant bitches

**Figure-2:**
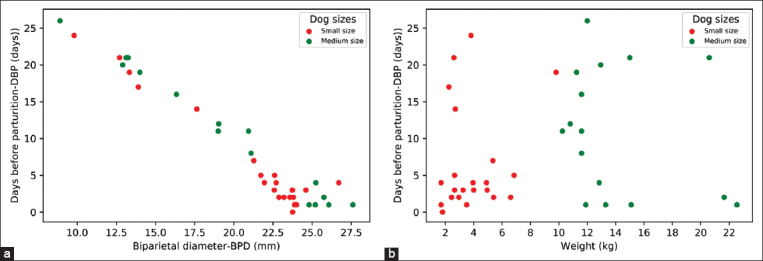
Scatter plot of testing datasets. Days before parturition (DBP) values acquired from testing datasets were plotted with their corresponding biparietal diameter (BPD) (a) and maternal weight (b). The red dots represented values acquired from small-sized bitches while the green ones represented those of medium-sized bitches.

According to the parturition date records, pregnant bitches in this study had DBPs ranging from 0 (parturition day) to 26 days. The BPD in small-sized bitches ranged from 9.20 to 26.80 mm (20.68±4.30 mm), whereas the BPD of medium-sized bitches ranged from 8.90 to 29.50 mm (30.00±5.63 mm). After data division into training and testing datasets, scatter plots show the relationships of BPD with DBP (Figure-2a) and maternal weight with DBP (Figure-2b) for the testing datasets.

### SVR parameter optimization

The parameters for the Rbf and linear and polynomial functions were successfully optimized. Continuous values in the determined ranges were tried in each kernel function to optimize the performance of each model in the training datasets. In the Rbf SVR, the regularization parameter (C) and kernel coefficient (gamma) were tuned. The linear SVR required only C parameter tuning, whereas the polynomial SVR required tuning of three parameters, the C parameter, independent term (coef0), and degree of the polynomial kernel function (degree). Further details and descriptions of these parameters can be found at https://scikit-learn.org/stable/modules/generated/sklearn.svm.SVR.html#sklearn.svm.SVR. All optimized SVR parameters used in each kernel function along with the SLR parameters for both small and medium dog sizes are shown in Table-2.

### Accuracy of SLR and SVR models for predicting DBP

**Figure-3:**
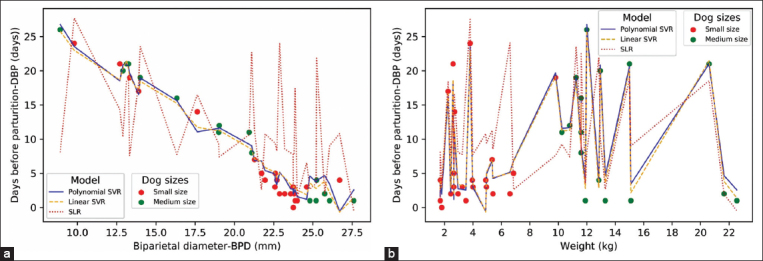
Simple linear regression (SLR) and support vector regression (SVR) plot. The predicted values generated by SLR, linear SVR, and polynomial SVR models were presented by different line types as the legend indicated. The radial basis function SVR was excluded for figure’s simplicity. The lines were plotted against scatter plots of days before parturition (DBP) versus biparietal diameter (BPD) (a) and DBP versus maternal weight (b). Erroneous DBP predicted by SLR in small-sized bitches (red dots) was obviously noticeable.

As shown in Table-3, the high R2, low MSE, and low MAE scores for the linear and polynomial SVRs were remarkable. Furthermore, the pairwise comparisons among models also revealed that the linear and polynomial SVRs had significantly better scores than the other models (p≤0.05) (Table-4). Comparisons between R2, MAE, and MSE scores for the SVR and SLR models showed that the linear and polynomial SVR models were the most accurate. To observe the outperformance of the linear and polynomial SVR models compared with that of the SLR model, the predicted DBP values acquired from all three models were plotted against the scatter plots of BPD versus DBP (Figure-3a) and maternal weight versus DBP (Figure-3b). Although deviations in the predicted DBP values using the SLR model were generalized along sorting BPD values (Figure-3a), obvious deviations were concentrated in small-sized bitches (<10 kg) (red dashed line in Figure-3b).

**Table 3:** R2, MAE and MSE scores.

Model	^a^R2 score	^b^MAE score	^c^MSE score
Small-sized bitch			
SLR	−0.17±0.58	2.50±0.24	8.02±1.60
Linear SVR	0.95±0.03	1.12±0.11	1.61±0.29
Polynomial SVR	0.95±0.03	1.13±0.11	1.68±0.30
Rbf SVR	0.85±0.07	1.50±0.15	2.91±0.47
Medium-sized bitch			
SLR	−0.18±0.59	2.49±0.23	8.03±1.52
Linear SVR	0.95±0.02	1.12±0.11	1.62±0.29
Polynomial SVR	0.95±0.03	1.13±0.11	1.69±0.29
Rbf SVR	0.85±0.07	1.50±0.14	2.91±0.47

a R2=Coefficient of determination,

b MAE=Mean absolute error,

c MSE=Mean squared error

**Table 4:** *Post hoc* Tukey HSD results.

Compared models		p-value		
	
Small-sized bitch		R2 score	MAE score	MSE score

Model 1	Model 2			
SLR	Linear SVR	0.001	0.001	0.001
SLR	Polynomial SVR	0.001	0.001	0.001
SLR	Rbf SVR	0.001	0.001	0.001
Linear SVR	Polynomial SVR	0.900	0.300	0.276
Linear SVR	Rbf SVR	0.001	0.001	0.001
Polynomial SVR	Rbf SVR	0.001	0.001	0.001

**Medium-sized bitch**		**R2 score**	**MAE score**	**MSE score**

**Model 1**	**Model 2**			

SLR	Linear SVR	0.001	0.001	0.001
SLR	Polynomial SVR	0.001	0.001	0.001
SLR	Rbf SVR	0.001	0.001	0.001
Linear SVR	Polynomial SVR	0.900	0.402	0.202
Linear SVR	Rbf SVR	0.001	0.001	0.001
Polynomial SVR	Rbf SVR	0.001	0.001	0.001

## Discussion

Limited DBP prediction accuracy for the SLR model using BPD is common among small- to medium-sized pregnant bitches. In agreement with previous reports [2,7,8], such inaccuracy was also noticeable in this study. These results support our objective to introduce SVR as an alternative regression model to improve the prediction of DBP (Figure-3). Although the acquired results strongly suggest that the linear and polynomial SVRs are better models than the SLR (Table-4), the SVR models could be further improved by the inclusion of larger BPD examination numbers acquired from more varied dog breeds.

Theoretically, the SVR model fitting relies on adjustment of the model’s error rate to contain as many DBP values as possible. The SVR model utilizes different kernel functions to transform the non-linear model into a linear one [15]. On the basis of such a concept, all observed DBP values would contribute to SVR fitting with fair weights correlated to one another. Such a feature is more effective in capturing confounding relationships among weights, BPD, and the predicted DBP than the routine dog size dependent SLR model. In support of this concept, all SVR models rendered better prediction accuracy than the SLR model, especially the linear and polynomial SVRs, which demonstrated the highest accuracy (Table-4). Similar to previous reports [2,7], the reduced prediction accuracy of the SLR model in small-sized pregnant bitches also occurred in this study (Figure-3). All acquired results suggest that both the linear and polynomial SVRs are suitable alternative regression models to predict DBP, and dog size categorization by maternal weight is not required in these models.

Accurate DBP prediction allows veterinary practitioners to make accurate decisions regarding critical delivery assistance in small- and medium-sized dog breeds with a high risk of dystocia [18]. Precise delivery predictions using linear and polynomial SVRs will contribute to better parturition management and an enhanced chance of survival for both mothers and pups. Although the major objective of this study was to verify SVR as an alternative tool for canine DBP prediction in small- to medium-sized dogs, more elaborate kernel functions and all dog sizes should be incorporated into a universal SVR model for DBP prediction in the future.

## Conclusion

Results from the current study demonstrate that the novel linear and polynomial SVR models accurately predict canine DBP in small- to medium-sized dogs. With ready-to-use statistical software available among computer platforms, the application of such a model has the potential to be implemented for future pediatric practices. With proper refinement, a user-friendly application could be developed for general practitioner use.

## Authors’ Contributions

TS and KC planned the study design, analyzed data, and drafted the manuscript. KM and SP collected the data. TS, KM, SP, and KC reviewed the manuscript. KC carried out technical coding correction. All authors read and approved the final manuscript.
